# Mapping PANSS factor dynamics: a novel framework for tracking therapeutic response in schizophrenia

**DOI:** 10.3389/fpsyt.2026.1864601

**Published:** 2026-09-24

**Authors:** Angelina I. Platova, Ekaterina V. Semina, Svetlana A. Zozulya, Anna S. Zinkevich, Yulia A. Chaika

**Affiliations:** Russian Mental Health Research Center, Moscow, Russia

**Keywords:** biomarkers, factor analysis, factor scores, neuroinflammation, PANSS, personalized medicine, schizophrenia spectrum disorders

## Abstract

**Objective:**

To study the factor structure of PANSS score dynamics in patients with schizophrenia spectrum disorders (SSD) and its relationship to individual patient characteristics.

**Methods:**

A single-center retrospective observational study was conducted. We analyzed medical records of patients with SSD from Russian Mental Health Research Center (Moscow, Russia), in whom neuroinflammation biomarkers had been monitored and item-level PANSS scores were available. Dynamics of individual PANSS items after therapy adjustment were examined via exploratory factor analysis (FA; n=182). The relationship between derived factor scores and potential predictors (clinical characteristics, therapy, sex, neuroinflammation markers) was assessed using multivariate regression (n=148).

**Results:**

We developed an algorithm for conducting and interpreting exploratory FA for ordinal psychometric test scores. A factor structure of PANSS dynamics has been developed that divides the regression of psychopathological features into three dimensions (directions): positive, negative, and affective symptoms. The initial PANSS scores, summed separately according to three dimensions of the factor structure, were found to have an effect on reducing negative symptoms. The positive influence of the female sex on the therapeutic response in negative and depressive dimensions was revealed. The use of antidepressants or anticonvulsants worsens the regression of positive symptoms, while the use of antidepressants reduces the regression of the depressive component. Cholinolytics as part of therapy are associated with regression of positive symptoms, and higher baseline values of blood leukocyte elastase activity in blood reduced the regression.

**Conclusions:**

The need to explore individual psychometric scale items via exploratory FA, along with the utility of factor scores as therapeutic response variables in psychiatry, is underscored. Identified patterns hold significance for personalized SSD treatment and warrant further investigation.

## 1 Introduction

Today, the Positive and Negative Syndrome Scale (PANSS) continues to serve as a principal instrument for evaluating treatment efficacy and therapeutic outcomes in patients with schizophrenia spectrum disorders (SSD). This scale, as well as contemporary approaches that cluster it into positive, negative, and general psychopathology domains, was developed on the basis of clinical-psychological symptomatology, distinct dimensions of which reflect underlying pathophysiological processes that cannot be measured directly. Currently, the international community has already accumulated a large number of studies devoted to the exploratory FA of the PANSS. While a pentagonal (five-factor) structure has become established for the initial stage of schizophrenia (1–3), this pattern may change as the disease progresses against the background of ongoing therapy. Thus, in a recent study of the PANSS structure in patients with schizophrenia, disorganization and cognitive components were distinguished, with the latter being identified as an additional, sixth factor (4). It has also been shown that, during treatment, individual symptoms of schizophrenia decrease independently of one another, with their regression proceeding along distinct trajectories, for example within separate clusters of positive and negative symptoms (5). Despite the substantial number of studies devoted to identifying and characterizing factors (12, 13), researchers in psychology and psychiatry have largely overlooked the factor-analytic examination of the dynamics of individual clinical-scale items. Yet the principal variable of therapeutic response in psychiatry is precisely the temporal change in psychometric scale scores (6, 7). We were unable to identify any studies that apply FA to the dynamics of individual PANSS item scores.

It is reasonable to assume that, in addition to the prescribed treatment, a range of individual patient characteristics also influence the therapeutic effect and may act as relevant predictors. The principal mathematical tool for identifying potential predictors that influence the magnitude and pattern of therapeutic response is regression analysis. However, the application of this approach in psychiatry is subject to a substantial limitation, namely the requirement that the dependent variable—i.e., the measure of therapeutic response—be continuous and approximately normally distributed (8, 9).

In psychiatry, therapeutic response is operationalized through changes in psychometric rating scales, which are inherently ordinal in nature (10). This limitation is further compounded by the multidimensional character of psychopathological symptomatology, with each patient exhibiting an individual profile of symptom expression and severity (6, 7).

The optimal solution to this problem may be to transform the space of collected variables into a smaller space of other variables while preserving information capacity, in other words, to use factor scores. Factor scores are continuous values with a symmetrical distribution, reflecting the magnitude of hidden factors. These latent factors, which cannot be measured directly, are the overall driving force and the cause of the joint change in the measured values, the scores of individual PANSS items (11). We did not identify any published studies in which factor scores derived from the PANSS were employed as the dependent variable in regression analyses.

The multi-stage nature of FA, along with the existence of numerous mathematical approaches for implementing each of its stages, contributes to the complexity of its application. The selection of statistical methods at each stage of FA is determined by the research objectives and the data type. To ensure appropriate application, comparability, and the potential integration of findings across studies, methodological standardization of FA in relation to psychometric scales is necessary (14). For example, some investigators apply FA, whereas others rely on principal component analysis (PCA); similarly, some adopt orthogonal rotation, while others choose oblique rotation (15, 16). The majority of studies rely on Pearson correlation matrices, even though the items of psychometric scales are inherently ordinal in nature. This treatment of ordinal data as continuous may further exacerbate bias in estimates, particularly when examining change over time or temporal dynamics in symptom scores (17, 18).

Modeling the dynamics of therapeutic response using linear multivariate regression is particularly important for the personalization of psychopharmacotherapy. Each patient is characterized by an individual profile of psychopathological symptoms, and the prescribed treatment should match this psychopathological profile in a “hand-in-glove” manner: it should primarily attenuate the most pronounced symptom dimensions, exert a lesser impact on less prominent dimensions, and avoid exacerbating other aspects of psychopathology. In light of this rationale, in our study the dependent variables in the regression analyses were factor scores representing the principal dimensions that capture the trajectory of psychopathological symptoms over time. The independent variables were correspondingly treated as predictors of the therapeutic response.

The therapeutic response is primarily determined by the status of the disease (clinical and psychopathological profile), as well as the composition of therapy and dosing regimen. Measurable characteristics of the disease include its duration, time from the manifestation and psychometric test scores. In addition, the therapeutic response may depend on the conditions of the formation and course of pathophysiological processes (the individual characteristics of the patients).

The search for biomarkers, measurable values that would reflect the pathophysiological profile of the disease, is one of the main problems of modern psychiatry.

A key area of research into the pathophysiology of schizophrenia in recent years has been neuroinflammation (19). It has previously been shown that levels of LE (leukocyte elastase), A1PI (alpha-1-proteinase inhibitor) and aAB-S100B (autoantibodies to astrocytic protein S100B) in patients with schizophrenia during a psychotic episode increase. In this connection, the level of immune system activation is observed several months before the onset of disease exacerbation (20). Currently, the autoimmune nature of neuroinflammation in schizophrenia is considered a promising area for future research. Antibodies against N-methyl-D-aspartate (NMDA) receptors, gamma-aminobutyric acid (GABA) receptor, neural cell adhesion molecule 1 (NCAM1), and neurexin 1 (NRXN1) have been found in schizophrenic patients (21). In addition, schizophrenia is associated with elevated levels of autoantibodies to myelin basic protein (aAB-MBP), which have proteolytic activity against myelin (22). Elevated levels of neuroinflammation biomarkers may be associated with resistance to treatment, more severe symptoms, and cognitive impairment in patients with SSD (23). Thus, neuroinflammation biomarkers can be considered predictors of the form and degree of therapeutic response.

The above allows us to consider the dynamics of SSD in terms of factor axes (dimensions), which makes it possible to study the dependence of the therapeutic response on drugs, the characteristics of the disease, and the patient themselves. Based on this concept, the research goals were formulated:

1. Construction of a factor model of PANSS score dynamics and obtaining factor scores.
2. Examination of the prognostic ability of individual patient characteristics (gender, therapy, duration of the disease, markers of neuroinflammation) in relation to reduction of the main dimensions of psychopathological symptoms.

## 2 Materials and methods

### 2.1 Study design

Single-center retrospective observational study.

### 2.2 Study population

Inclusion criteria: patients observed at the Russian Mental Health Research Center (Moscow, Russia), either during hospitalization or on an outpatient basis, in 2017–2018, with diagnoses within the F20–F29 group (schizophrenia, schizotypal and delusional disorders), for whom serum neuroinflammation markers were obtained and item-level PANSS scores were available both at admission and at discharge following pharmacotherapy adjustment (n = 182). Methods for handling missing data were not applied, as both the FA and the regression analysis were conducted on complete cases with no missing observations for any of the variables.

A Russian-language 30-item version of the Positive and Negative Syndrome Scale (PANSS) was used for the assessment (based on S.P. Kay, L.A. Opler, and A. Fitzbane, 1987) (24, 25).

Exclusion criteria: presence of concomitant diseases and chronic conditions that may increase markers of systemic inflammation (cardiovascular disease, type 2 diabetes mellitus, oncology, chronic kidney disease, fatty liver disease, autoimmune diseases, chronic respiratory diseases, inflammatory diseases of the gastrointestinal tract, hyperuricemia, metabolic syndrome, etc.).

### 2.3 Predictors

Predictors included initial nosological status (time from onset, duration of illness, initial assessments of scores for PANSS separate items, summed up according to established factors), the presence in the therapeutic schema of antidepressants (AD), anticonvulsants (AK), anxiolytics (AN), lithium, anticholinergic drugs (0-No, 1-Yes), and other patient characteristics. These characteristics included gender and biomarkers: LE and A1PI activity, aAB-S100B and aAB-MBP levels. Both baseline biomarker levels and their dynamics during treatment were assessed. The regression analysis included data from patients who had observations for all predictor variables (n=148).

The biomarker levels in patients’ plasma samples have received from database on samples included in the Biomaterial Collections Fund “NeuroResource”, registered at the Russian Mental Health Research Centre (Moscow, Russia, https://nrc.ncpz.ru/).

### 2.4 Statistical analysis

Exploratory FA included the following stages: 1) Construction of a correlation matrix using Spearman’s method; 2) Factors were identified using FA, and their optimal number was determined by parallel analysis; 3) Analysis of the factor correlation matrix, selection of the rotation method, and the initial rotated factor solution; 4) Simplifying and stabilizing the factor structure; 7) Computation of factor scores using regression-based methods.

The methods and approaches used at each stage of the FA were determined by the results obtained at the preceding stage (26); therefore, we present a detailed, stepwise description and justification of the methodology in the Results section.

Next, factor scores were included as dependent variables in regression analysis, which assessed the predictive ability of individual patients’ characteristics. Baseline scores are often strongly associated with subsequent change (27). Therefore, in the regression analysis, baseline scores for each factor were included as predictors (for a detailed description of the methodology, see Section 3.3).

### 2.5 Software

R v. 4.3.1 (packages readxl, xlsx, dplyr, tidyverse, stats, corrplot, ggcorrplot, psy, psych, broom, car, GGally, DescTools).

## 3 Results

### 3.1 Key characteristics of patients

The FA included data from all patients with SSD who met the inclusion/exclusion criteria, whom measured markers of neuroinflammation in the blood at 2017-2018 (n=182). The regression analysis included data from patients with all observations for all predictor variables (n=148). The duration of observation for patients in the study was 4.1 (3.3-5.9) (median, 1st and 3rd quartiles) weeks. The most common diagnosis among patients was schizophrenia (62.64%) (**Table 1**).

**Table 1:** Characteristics of patients.

Predictors	Values	All included patients (n=182)		patients with observations for all predictors (n=148)	
		n	Fraction, %	n	Fraction, %
Gender	Male (0)	70	38.46%	56	38.10%
	Female (1)	112	61.54%	91	61.90%
Diagnosis	F 20 Schizophrenia	114	62.64%	90	60.81%
	F 21 Schizotypal disorder	37	20.33%	33	22.30%
	F 23 Acute and transient psychotic disorders	3	1.65%	3	2.03%
	F 25 Schizoaffective disorders	28	15.38%	22	14.86%
Category	Inpatients	140	94.59%	114	77.03%
	Outpatients	42	28.38%	34	22.97%
AD therapy	Yes (1)	80	44.69%	66	44.59%
	No (0)	99	55.31%	82	55.41%
AK therapy	Yes (1)	49	27.37%	40	27.03%
	No (0)	130	72.63%	108	72.97%
AN therapy	Yes (1)	16	8.94%	14	9.46%
	No (0)	163	91.06%	134	90.54%
Li therapy	Yes (1)	8	4.47%	6	4.05%
	No (0)	171	95.53%	142	95.95%
Descriptive statistics of quantitative variables (patients with observations for all predictors, n=148)					
Predictors	Mean	SD	Median	1st - 3rd quartiles	
Number of drugs	2.8	1	3	(2; 3)	
Number of drug groups	1.9	0.7	2	(1; 2)	
Duration of the disease	106.9	92	78	(36; 159)	
Time from the beginning of the disease manifestation	43.3	76.9	8	(3; 48)	
LE	243.5	35.1	242	(218.2; 270.6)	
A1PI	45.3	9.2	45.8	(39.4; 52.8)	
aAB-S100B	0.8	0.1	0.74	(0.7; 0.8)	
aAB-MBP	0.8	0.1	0.75	(0.7; 0.9)	
d_LE (dynamics)	-36.0	94.6	-6.5	(-45.9; 17.9)	
d_A1PI (dynamics)	-6.4	18.7	-3.3	(-13.3; 5.9)	
d_ aAB-S100B (dynamics)	-0.1	0.3	-0.03	(-0.1; 0)	
d_ aAB-MBP (dynamics)	-0.1	0.3	-0.03	(-0.1; 0)	

SD, standard deviation; “d_”, the dynamics of biomarkers was calculated as “the value after treatment – the baseline level”.

### 3.2 Factor analysis

#### 3.2.1 Creating a correlation matrix

FA can be performed on correlation or covariance matrices. A covariance matrix requires a normal distribution of the initial variables. The values of the dynamics of PANSS separate items are ordinal, contain many zeros and do not obey the normal distribution law. Therefore, to obtain the correlation matrix, we used Spearman method, which ranks zero values, and FA have performed strictly on data of this correlation matrix (**Figure 1**).

**Figure 1:**
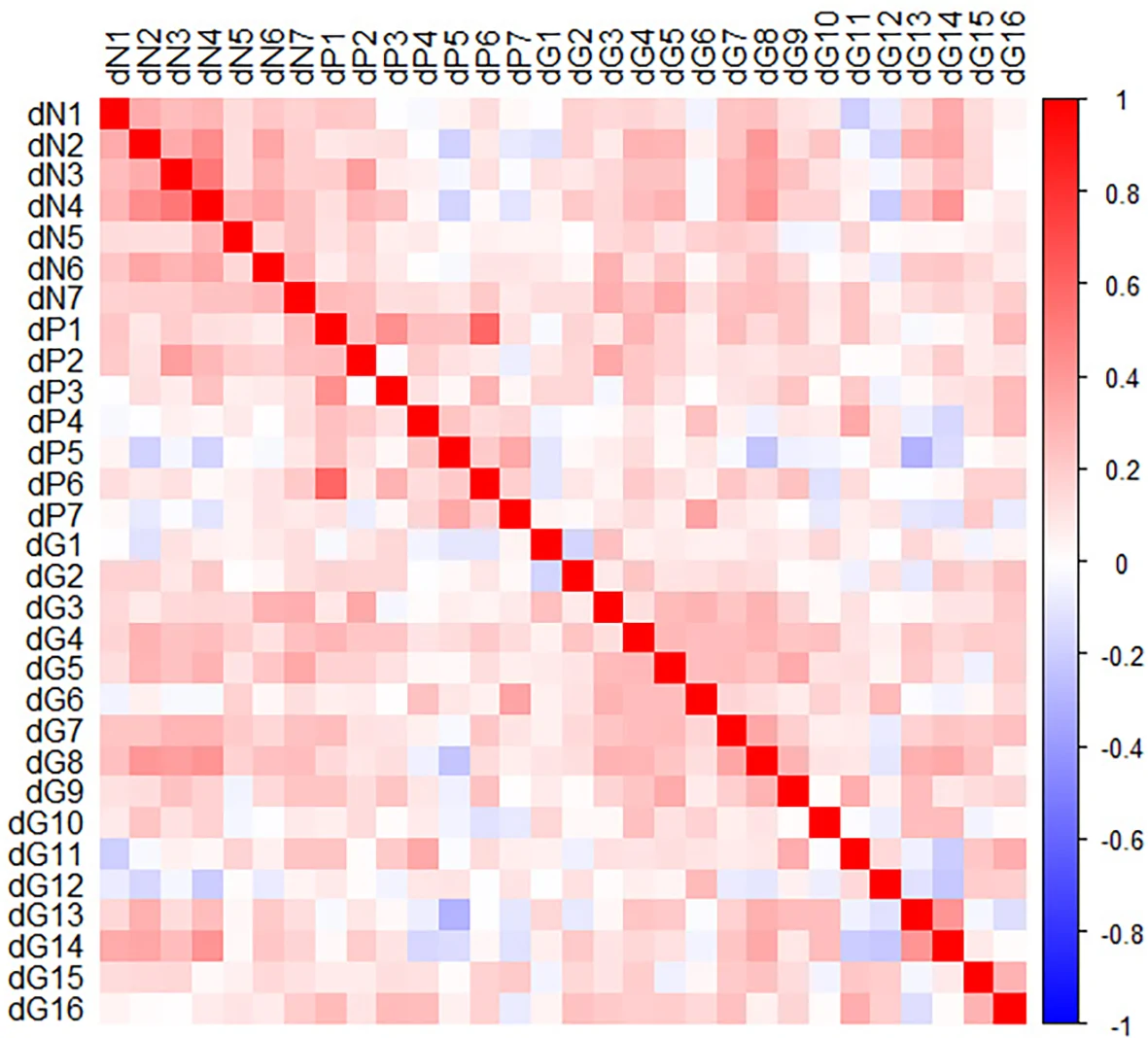
Correlation matrix of the dynamics of separate PANSS scale tests. D, dynamics; N, negative; P, positive; G, general psychopathology items. The color gradient reflects the strength of the correlation.

The suitability of the obtained matrix for FA was confirmed using Kaiser-Meyer-Olkin criteria (0.73) and Bartlett’s test of sphericity (p<0.0001).

#### 3.2.2 The choice of factor extraction method and the selection of the number of factors

The choice of factor extraction method depends on the research objectives, the type of data and the nature of its distribution, as well as the sample size. Two general approaches are often distinguished here: PCA and FA itself. PCA treats components as linear combinations of variables, while FA solves the inverse problem: hidden factors determine the observed variables, taking into account the proportion of unexplained variation. PCA is suitable for simple data reduction (number of variables) and usually involves the use of continuous variables. Therefore, FA is most preferable for analyzing ranked data, in particular the ordinary least squares method, which provides reliable results without assumptions about distribution. This method is sensitive to weak factors and less biased in terms of factor loadings (28).

The selection of the number of factors can be based on Kaiser rule, which recommends selecting factors with eigenvalues greater than 1. A more reasonable approach is to select the number of factors above the inflection point on the graph, which reflects the first sharp slowdown in the decline of the eigenvalues (29, 30). However, the most accurate method for determining the number of factors is parallel analysis, which generates random samples (31). The arrow in the figure indicates the point at which the actual eigenvalues exceed the medians of the simulated samples, which cuts off the three factors shown by triangles (**Figure 2**).

**Figure 2:**
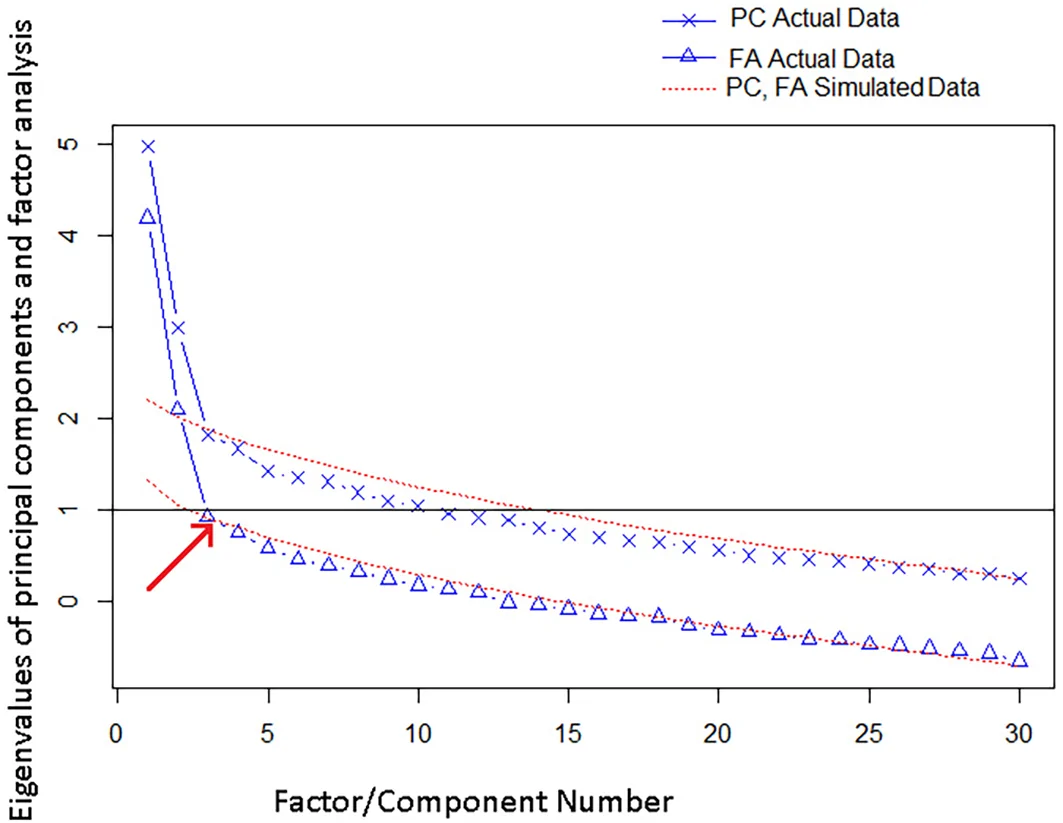
Chart of the dependence of the eigenvalues of the factors/components on their number in their order of extraction. 0Y axis shows the eigenvalues, 0X-axis shows the number of factors/components. PC, principal component analysis; FA, factor analysis; Actual Data, sample data; Simulated Data, simulated samples. In parallel analysis, 1000 iterations were performed.

#### 3.2.3 Analysis of the factor correlation matrix, selection of the rotation method, and the initial rotated factor solution

The initial unrotated factor solution provides an understanding of the relationship between factors and determines the choice of rotation method. We can see that one of the factors correlates with two others, with a correlation coefficient (r) > 0.32 (**Table 2**).

**Table 2:** The correlation matrix of the primary factorial solution.

Factors	Factor 1	Factor 2	Factor 3
Factor 1	1	0.1335766	**0.3528299**
Factor 2	0.1335766	1	**0.3904689**
Factor 3	**0.3528299**	**0.3904689**	1

Correlation coefficients greater than 0.32 are indicated in bold.

The reason for the correlation of factors is that some variables depend on more than one factor. The choice of factor axis rotation method depends on the research objectives (e.g., structural analysis, creation of a new scale, etc.). Rectangular rotation is appropriate when all coefficients r <0.32 (the total variance between factors is less than 10% (0.32^2^). It simplifies interpretation and has clear advantages if factor scores are to be used as dependent or independent variables in another analysis (32). We applied orthogonal rotation using the varimax method, which fixes structures where each variable has a high loading on one factor and close to zero on others (33, 34). Since orthogonal rotation gives less accurate factor scores if the factors correlate (35), variables that depend on more than one factor should be excluded from further analysis. The rotated factor solution is shown in **Table 3**.

**Table 3:** Characteristics of the factor structure and separate variables (dynamics of PANSS items).

The structure matrix (after rotation)								
Items dynamics	PANSS items	F1	F2	F3	h2	com	Baseline	Dynamics
dP1	Delusions	0.15	**0.72**	0.19	0.583	1.2	4 (3; 4)	-2 (-3; -1)
dN4	Passive/apathetic social withdrawal	**0.7**	0.11	-0.04	0.498	1.1	3 (2; 4)	-1 (-1; -1)
dP6	Suspiciousness/Persecution	0.08	**0.64**	0.08	0.428	1.1	3 (2; 4)	-1 (-2; 0)
dN2	Emotional withdrawal	**0.63**	0.07	-0.08	0.414	1.1	3 (3; 4)	-1 (-1; -1)
dG14	Poor impulse control	**0.6**	-0.03	-0.2	0.396	1.2	3 (2; 3)	-1 (-2; 0)
dG13	Disturbance of volition	**0.55**	-0.23	-0.01	0.354	1.4	4 (2.75; 4)	-2 (-2; -1)
dG8	Uncooperativeness	**0.57**	0.07	0.14	0.352	1.1	3 (3; 4)	-1 (-2; -1)
dG5	Mannerisms and posturing	**0.40**	0.08	**0.39**	0.317	2.1	3 (3; 4)	-1 (-1; 0)
dN7	Stereotyped thinking	**0.37**	0.18	**0.37**	0.304	2.4	4 (3; 4)	-1 (-1; -1)
dG3	Guilt feelings	0.29	-0.04	**0.46**	0.302	1.7	3 (3; 4)	-1 (-1; 0)
dN3	Poor rapport	0.51	0.17	0.05	0.297	1.2	3 (3; 4)	-1 (-2; -1)
dG6	Depression	0.02	-0.01	**0.54**	0.297	1	2 (1; 3)	-1 (-1; 0)
dN1	Blunted affect	0.48	0.17	-0.07	0.268	1.3	3 (3; 4)	-1 (-1; -1)
dG4	Tension	0.33	0.25	0.28	0.256	2.8	4 (3; 4)	-1 (-2; -1)
dG16	Active social avoidance	0.06	0.32	0.36	0.236	2	3 (2; 4)	-1 (-1; -1)
dG7	Motor retardation	0.39	0.2	0.22	0.235	2.1	3 (3; 4)	-1 (-2; -1)
dP3	Hallucinatory behavior	0.13	**0.46**	0.07	0.23	1.2	2 (1; 4)	-1 (-2; 0)
dG11	Poor attention	-0.01	0.2	**0.41**	0.207	1.5	3 (2; 3.25)	-1 (-2; -1)
dG9	Unusual thought content	0.28	0.21	0.27	0.196	2.8	4 (3; 4)	-2 (-2; -1)
dN6	Lack of spontaneity and flow of conversation	0.42	0.05	0.12	0.193	1.2	3 (2; 4)	-1 (-1; 0)
dG12	Lack of judgment and insight	-0.23	0.08	**0.35**	0.183	1.8	1 (1; 2)	0 (-1; 0)
dP5	Grandiosity	-0.23	**0.32**	0.13	0.175	2.2	1 (1; 1)	0 (0; 0)
dP4	Excitement	-0.07	0.26	0.31	0.168	2	3 (2; 3)	-1 (-2; -0.75)
dP2	Conceptual disorganization	0.32	0.1	0.22	0.159	2	3 (3; 4)	-1 (-1; -1)
dP7	Hostility	-0.17	0.15	0.3	0.144	2.1	2 (1; 3)	0 (-1; 0)
dG1	Somatic concern	0.14	-0.19	0.22	0.105	2.7	2 (1; 3)	0 (-1; 0)
dN5	Difficulty in abstract thinking	0.22	0.04	0.23	0.102	2.1	3 (2; 3)	-1 (-1; 0)
dG2	Anxiety	0.12	0.3	0	0.101	1.3	1 (1; 1)	0 (0; 0)
dG10	Disorientation	0.25	-0.16	0.06	0.092	1.9	1 (1; 2)	0 (-1; 0)
dG15	Preoccupation	0.05	0.25	0.17	0.091	1.8	1.5 (1; 2)	0 (-1; 0)
Factor model summaries								
SS loading (sum of squared loading)		3.71	2.04	1.94				
Proportion of Variance		12%	7%	6%	Total 25% (12 + 7 + 6) variations of all variables can be explained by a factor model			
Proportion Explained		48%	27%	25%				

*median (1st and 3rd quartiles). Factor loadings: F1, factor of dynamics of the negative symptoms; F2, factor of dynamics of the positive symptoms; F3, factor of dynamics of the affective disorders (or affective dimension). The factor loads making the greatest contribution to the corresponding variables are highlighted by the fill. Variables with significant correlations (r above 0.32) with any kind of factors are highlighted in bold.

SS loading (sum of squared loading), the sum of the squares of each factor loadings; proportion of variance, the proportion of variance of variables, explained by each factor; proportion explained, the amount of total data variance accounted for by a specific factor in factor analysis; h2, communality; com, Hoffman complexity index, determines the scheme of loading a variable with factors is how complex, the higher the value, the variable is more complex.

Structural analysis shows that PANSS dynamics during treatment occur in the following dimensions: a decrease in negative, positive, and affective symptoms. The same (positive) sign of significant factor loadings (>0.32) for all variables reflects the unidirectional nature of their dynamics.

The factor of dynamics of the negative symptoms (F1) also increases impulse control, alleviates disturbance of volition, improves cooperativeness, and reduces motor retardation.

The reduction of positive symptoms (F2) appears like the concomitant reduction in the severity of hallucinations, suspiciousness, delusions of grandeur, and delusions.

The relief of affective disorders (F3) is associated with items “Lack of judgment and insight”, “Active social avoidance” and “Stereotyped thinking”. The last item has the same load on the factor of dynamics of the negative symptoms. In addition, item “Mannerisms and posturing” shows synchrony with the treatment of negative symptoms, and the “Tension” test evenly reflects all three dimensions.

#### 3.2.4 Simplifying and stabilizing the factor structure

We simplified and stabilized the factor structure using the following procedure: 1) determining the threshold value for factor loadings; 2) removing variables with loadings above the threshold value by more than 1 factor; 3) removing variables with a difference in loadings of less than 0.1 (36); 4) removing variables with all loadings below the threshold value; 5) assessing the remaining variables to determine whether their communalities exceed the specified threshold value (0.2) (36); 6) assessing the internal consistency of the factor model.

The sequence of variable removal within each stage was determined by the complexity coefficient, in descending order.

*The threshold value for factor loadings* is determined by the research objectives. The following cut-off limits have been adopted: 0.32 (poor, corresponding to 10% variation), 0.45 (fair, 20%), 0.55 (good, 30%), 0.63 (very good, 40%), or 0.71 (excellent, 50%) (32). It is important to note that the dynamics of individual PANSS items have a much smaller spread than the initial scores for these items: the multidimensional correlation ellipse of the initial test scores transforms into a sphere when assessed in terms of dynamics due to the reduction in spread. **Table 3** shows a noticeable trend that the decrease in the commonality of individual variables reflects a reduction in the dynamics of their scores and a tendency for their median to approach zero. Therefore, in our study, the cutoff point for factor loadings was set at 0.32.

*Variables* were *removed* step by step as the complexity index decreased. *The commonality* of the *final* structure of all variables was not less than 0.2 (**Table 4**).

**Table 4:** The results of the factor analysis after simplification of the structure.

Factor loading matrix after simplification of the factor structure (Varimax rotation)									
Item		Value			F1	F2	F3	h2	com
dN4		Passive/Apathetic Social Withdrawal			**0.69**	0.14	-0.06	0.50	1.1
dN2		Emotional Withdrawal			**0.65**	0.04	0.03	0.43	1
dG8		Uncooperativeness			**0.59**	0.11	0.23	0.41	1.4
dG14		Poor Impulse Control			**0.6**	-0.02	-0.1	0.37	1.1
dN3		Poor Rapport			**0.51**	0.18	0	0.29	1.2
dG13		Disturbance of Volition			**0.49**	-0.11	-0.02	0.26	1.1
dN1		Blunted Affect			**0.47**	0.14	0.01	0.24	1.2
dP3		Hallucinatory Behavior			0.1	**0.48**	-0.01	0.24	1.1
dP1		Delusions			0.08	**0.86**	0.1	0.75	1
dP6		Suspiciousness/Persecution			0.04	**0.66**	0.09	0.45	1
dG6		Depression			-0.01	0	**0.76**	0.57	1
dP7		Hostility			-0.17	0.11	**0.47**	0.26	1.4
dG3		Guilt Feelings			0.24	0.03	**0.39**	0.21	1.6
Factor model summaries									
SS loading (sum of squared loading)					2.4324	1.5164	1.0367	-	
Proportion of Variance					19.0%	12.0%	8.0%	-	
Proportion Explained					49.0%	31.0%	20.0%	-	
Factor correlation matrix*									
	Factor 1		Factor 2	Factor 3					
Factor 1	1		0,1670583	0,03946236					
Factor 2	0,16705828		1	0,10822875					
Factor 3	0,03946236		0,1082288	1					

Factor loadings: F1 - factor of dynamics of the negative symptoms, F2 - factor of dynamics of the positive symptoms, F3 - factor of dynamics of the affective disorders (or affective dimension). The factor loadings making the greatest contribution to the corresponding variables are highlighted by the fill. Variables with significant correlations (r above 0.32) with any kind of factors are highlighted in bold. SS loading (sum of squared loading), the sum of the squares of each factor loadings; proportion of variance, the proportion of variance of variables, explained by each factor; proportion explained, the amount of total data variance accounted for by a specific factor in factor analysis; h2, communality; com, Hoffman complexity index, determines the scheme of loading a variable with factors is how complex, the higher the value, the variable is more complex.

An orthogonal rotation of the factor axes preserves the factor-correlation matrix, leaving the correlations among factors unchanged (37).

After simplification of the factor structure, the correlations among factors decreased substantially (**Table 4**), which justified the use of varimax rotation.

Internal consistency was assessed by calculating Cronbach’s alpha (based on Spearman’s correlation), which reflects the reliability of the set of scale items, showing how well the items correspond to the same basic construct. This coefficient was 0.78 for the “Negative Block Regression” factor, 0.71 for the “Positive Block Regression” factor, and 0.51 for the “Affective Block Regression” factor. For the first factors, the consistency of the scale is acceptable, while for factor 3, it is weak. This may be due to the poor representation of affective symptoms in the PANSS, as well as the heterogeneity of its composition (depression, hostility, and guilt).

#### 3.2.5 Computation of factor scores

Factor scores were computed using a regression-based approach. The main critic of FA, Tabachnik B., points out that this method is preferable in all cases due to its simplicity of interpretation, except when uncorrelated factor scores are required, in which case the Anderson-Rubin method is preferable (32). Factor scores reflect the value of the latent factor, and this value is the sum of the contributing variables with loadings as coefficients. In the **Table 4**, we see that all weighted factor loadings have a positive sign. This means that the higher the factor scores, the greater the values of the variables loading this factor, and *vice versa*. In other words, greater symptom regression corresponds to lower factor scores for the respective dimension.

### 3.3 Regression analysis of factor scores as therapeutic outcome variables

The individual factor scores obtained represent the degree of symptom regression for each patient in the following domains (factors): negative symptoms (F1), positive symptoms (F2), and affective symptoms (F3) (**Table 4**). These values were considered as dependent variables in the study of the predictive ability of individual patient characteristics.

The dynamics of psychopathological patterns are primarily determined by therapy and initial conditions (38, 39). These values were included in the regression model in the first step of analysis. All patients received antipsychotics (AP), so their influence was incorporated into the model by adding a constant (intercept). The therapy took into account the total number of psychotropic drugs received (except for anticholinergics), the number of drug groups (AP, AD, AK, anxiolytics, lithium salts), as well as each of these groups separately. In psychiatry AK and lithium salts are used as normothymics, but since these drugs have fundamentally different mechanisms of action, they were considered as separate groups. The initial conditions were understood as the initial PANSS test scores that most clearly reflected certain factors (F1sum, F2sum, F3sum). For example, the F2s value was calculated as the sum of PANSS scores for the following items: P3 (Hallucinatory behavior), P1 (Delusions), P5 (Grandiosity) and P6 (Suspiciousness) (**Table 4**).

The following predictors were then added to the model: time from disease onset and disease duration (in months), neuroinflammation indicators (baseline level at the time of consultation/hospital admission, as well as dynamics during treatment), and gender. These predictors were added to the regression model in the second step of the analysis. The assessment of assumptions and the core principles guiding the interpretation of the regression analyses were conducted in accordance with the foundational textbook by Draper and Smith (1966) (40).

The characteristics of the final models are shown in **Table 5**. Since different predictors were measured in incomparable units, their influence can be compared by comparing standardized coefficients. It should be noted that lower factor scores correspond to greater symptom regression.

**Table 5:** Results of multiple regression analysis.

Model	Unstandardized coefficients		Standardized coefficients	t (Student test)	p-value	95% CI for B	
	B	SE	Beta			Lower limit	Upper limit
Dependent variable F1, adjusted R2 = 0,685, p-value of the F-test <0,001							
Constant (intercept)	10.545	0.359		40.304	<0.001	0.835	20.254
Gender	-0.539	0.120	-0.284	-40.477	<0.001	-0.777	-0.301
F1sum	-0.100	0.011	-0.575	-90.214	<0.001	-0.121	-0.079
F2sum	0.042	0.018	0.146	20.285	0.024	0.006	0.077
F3sum	0.073	0.026	0.172	20.781	0.006	0.021	0.125
Dependent variable F2, adjusted R^2^ = 0,537, p-value of the F-test <0,001							
Constant	-10.220	0.442		-20.762	0.006	-20.093	-0.347
Antidepressants	0.632	0.129	0.364	40.915	<0.001	0.378	0.886
Anticonvulsants	0.494	0.141	0.252	30.494	0.001	0.214	0.773
LE (baseline)	0.004	0.002	0.172	20.377	0.019	0.001	0.008
Anticholinergics	-0.371	0.130	-0.213	-20.861	0.005	-0.627	-0.115
Dependent variable F3, adjusted R^2^ = 0,326, p-value of the F-test <0,001							
Constant (intercept)	-0.065	0.111		-0.590	0.556	-0.284	0.154
Gender	-0.326	0.130	-0.200	-20.517	0.013	-0.582	-0.070
Antidepressants	0.456	0.126	0.286	30.610	<0.001	0.207	0.706

F1s, F2s, and F3s are the sum of baseline PANSS items scores contributing to factors F1, F2, and F3, respectively; adjusted R2 – the coefficient of determination adjusted by the Wherry formula, reflecting the proportion of variability of the dependent variable explained by the model; F-criterion – Fisher’s criterion, reflecting the significance of the models as a whole.

Therefore, a negative sign of the predictor coefficients means a favorable effect, and a positive sign means the unfavorable. A negative intercept sign in all models means a basic regression of symptoms, which we interpret as the therapeutic effect of AP (**Table 5**).

As shown in **Table 5**, the regression of negative symptoms (F1) is positively associated with their baseline severity (F1sum) and with female gender (coded as “1” in the model), and is negatively associated with the F2sum and F3sum values. The regression of positive symptoms (F2) is associated with the addition of anticholinergic agents to the treatment regimen, which likely reflects an increased dopaminergic receptor load induced by AP therapy. The course of positive symptoms appears to be unfavorably influenced by the prescription of AK and/or AD. There is also a clear negative association between the baseline level of LE and the subsequent regression of positive symptoms. In the regression model, AD prescription was associated with a less favorable trajectory of the “Regression of affective symptoms” factor (F3), whereas female sex, in contrast, was associated with greater improvement in this dimension. The intercept term in this model was not statistically significant (p = 0.556), which may suggest the absence of a distinct independent effect of AP treatment on F3 (**Table 5**).

No influence of anxiolytics and lithium preparations on factor scores was found, which is associated with their weak representation in the sample (9.46% of patients received anxiolytics, 4.05% received lithium preparations (**Table 1**).

## 4 Discussion

In this study, we examined and proposed an optimized algorithm of exploratory FA (not based on PCA), implemented using ordinary least square method, for the evaluation of psychometric scales.

PCA primarily targets the decomposition of variance, which is not fully appropriate for nonparametric, ordinally scaled scores. In contrast, our FA was conducted exclusively on a correlation matrix comprising Spearman rank correlations (17), methodological approach that, to our knowledge, has not been explicitly employed or discussed in previous PANSS factor-analytic studies (18).

Most studies examining the factor structure of the PANSS have relied on PCA, typically in combination with varimax rotation (15, 16), and this specific pairing has been substantially criticized in the mathematical and statistical literature (41).

In contrast, the combination of FA with ordinary least squares estimation and varimax rotation employed in the present study offers distinct advantages for psychometric scale data, particularly in terms of robustness with ordinal variables and the attainment of an interpretable simple structure (42).

The proposed algorithm for simplifying and stabilizing the factor solution provides an efficient and straightforward means of refining and clarifying the underlying factor structure.

Key contribution of the present study is the analysis of the factor structure of PANSS dynamics, which captures treatment-related changes along empirically derived positive, negative, and affective dimensions rather than relying solely on traditional subscale sums.

Furthermore, we demonstrate that factor scores obtained from exploratory FA can serve as informative continuous outcome variables in regression models, enabling a more nuanced quantification of therapeutic response in SSD.

To our knowledge, no published studies have examined the factor structure of the dynamics of individual PANSS items over time. Likewise, we did not identify any publications in which PANSS factor scores were used in regression analyses, with the exception of a single study in which PANSS factor scores were included as predictor variables rather than outcomes (43). Therefore, a direct comparison of our findings with those reported in previous studies is not feasible.

Another novel aspect of this work is the combined analysis of PANSS dynamics factor scores and neuroinflammation biomarkers, which provides an integrated framework for linking psychopathological change to putative pathophysiological processes.

The main limitation of this study appears to be its methodological design, which is retrospective, observational, single-center, and non-randomized. An additional important limitation concerns the pharmacological heterogeneity of the sample: participants received diverse combinations of APs, ADs, AKs, and anticholinergic agents, rendering the interpretation of treatment–response associations particularly challenging.

In this study, our objective was to propose a methodological framework that enables the effective use of real-world clinical data. With increasing sample size (for example, through the aggregation of data from multiple observational studies) and subsequent harmonization of the dataset to conform to a balanced factorial design, this approach may facilitate the identification of clinically meaningful predictors and refine the interpretation of relationships between separate drugs, individual patient characteristics, and clinical outcomes, thereby supporting the subsequent prediction of therapeutic response.

To confirm the clinical relevance of the potential predictors identified, we plan a future study in which confirmatory FA will be conducted as a subsequent stage of factor modeling, with validation performed in an independent sample (44–47). For exploratory FA, a commonly recommended rule of thumb is that the number of observations should be approximately five times the number of variables (i.e., at least 30 × 5 = 150 participants). In the present study, FA was exploratory, and therefore our sample size can be considered adequate (n = 182). By contrast, confirmatory FA generally requires substantially larger samples, which should be taken into account in future studies aiming to confirm the factor structure using confirmatory FA (11, 45, 47). There is no strict “one-size-fits-all” formula for calculating sample size in confirmatory FA. Taking into account the expected factor loadings (≥ 0.32), communality values of at least 0.20, the presence of three latent factors with 13 observed variables, and a desired statistical power of 80% (the chi-square test, Monte-Carlo simulations), the required sample size for confirmatory FA can be estimated at approximately 300 patients (48–50).

### 4.1 The nature of latent factors underlying the dynamics of PANSS item scores

In line with current neurobiological models, the reduction of negative symptoms in schizophrenia spectrum disorders may be related to pharmacological mechanisms involving D_3_-dopamine receptor blockade and, potentially, α_2_-adrenoceptor blockade (51).

The core of the dynamics of positive symptoms was the regression of hallucinations, delusions, and suspiciousness, which is plausibly linked to dopamine D_2_-receptor blockade in mesolimbic pathways. At the same time, antagonism at M_1_-, M_4_-muscarinic and α_1_-adrenergic receptors, although primarily associated with side-effect profiles and modulation of arousal, may exert additional, potentially bidirectional influences on the course of positive symptoms (52).

The regression of depressive and affective symptoms in SSD is thought to rest on a multicomponent neurobiological basis - including alterations in the hypothalamic–pituitary–adrenal (HPA) axis, changes in brain-derived neurotrophic factor (BDNF), and disturbances in neuroplasticity - rather than solely on the binding profile of individual receptors. Contemporary models also implicate multiple neurotransmitter systems, notably glutamatergic NMDA (N-methyl-D-aspartate) receptors, various serotonin receptor subtypes (e.g., 5-HT_2_), and dopaminergic D_2_/D_3_ receptors, whose functional effects depend on ambient dopamine levels (53–55).

### 4.2 Interpretation of the potential predictors of factor scores reflecting PANSS dynamics

#### 4.2.1 The effect of gender

Emerging evidence indicates that women with SSD often exhibit a more favorable response to antipsychotic treatment, frequently at lower doses, a phenomenon that has been tentatively attributed to estrogen-related modulation of dopaminergic and other neurotransmitter systems. In addition, women tend to show higher AP plasma concentrations and a greater burden of adverse effects (56, 57), which is consistent with sex-related differences in pharmacokinetics and pharmacodynamics. Taken together, these factors provide a plausible explanation for the more pronounced therapeutic response observed in women on the negative and affective symptom dimensions.

#### 4.2.2 The effect of the initial clinical status

A higher baseline score on the negative symptom block (F1sum) was associated with a greater subsequent reduction in negative symptoms, which is consistent with the notion that higher initial severity confers greater potential for this factor. In contrast, higher baseline scores on the positive (F2sum) and affective (F3sum) blocks were associated with a less favorable therapeutic response on factor descending the negative symptoms (F1). This pattern may suggest increased resistance of negative symptoms to treatment in patients presenting with more pronounced positive and affective psychopathology.

#### 4.2.3 The effect of therapy

The negative impact of AD and AK on the regression of positive symptoms, and of AD on affective symptoms, underscores the need for careful justification of their use in this population. This observation is consistent with current clinical recommendations, which do not regard antidepressants as drugs of choice for the treatment of depressive symptoms in patients with SSD (54). Moreover, the adverse association between AD treatment and improvement in the affective domain may point to specific features of the pathogenesis of affective symptoms in SSD, warranting further targeted investigation.

On the other hand, each drug has its own individual receptor affinity profile. Therefore, instead of conditionally dividing therapy into pharmacological groups, in future work, it may become possible to quantify and consider the overall pharmacological load on specific receptor systems (by taking into account molar doses and receptor affinity). This may will make it possible to assess the relationship between the activity of individual receptor types and the therapeutic response and provide practical recommendations for selecting the most optimal combinations of drugs to achieve the maximum therapeutic result.

Also, it is interesting to note that in the treatment of SSD, increased self-control of impulsive behavior (G14, Poor impulse control in Factor 1) is associated with a reduction in negative symptoms, and a decrease in hostility (P7 in Factor 3) occurs in conjunction with a decrease in manifestations of depression and anxiety (**Table 4**). The combination of impulsivity and aggression is a serious therapeutic problem that requires treatment approaches targeting both of these components.

#### 4.2.4 The effect of neuroinflammation markers

The dynamics of neuroinflammation markers did not show predictive properties, which can be explained by the delayed relationship between their blood levels and the intensity of neuroinflammation and psychopathological picture.

The negative effect on the regression of positive symptoms (on Factor 2) of the initial level of LE activity is may be explained by the intensification of neuroinflammatory processes. It is also important to discuss the fact that the prediction of factor scores of PANSS dynamics without taking into account therapy did not identify LE activity as a significant predictor (p=0.094), i.e., the effect of therapy camouflaged the influence of this biomarker. This may also explain the lack of a significant predictive effect in other markers of neuroinflammation - A1PI activity, as well as aAB-S100B and aAB-MBP. The results obtained indicate the importance of taking therapy into account when studying the predictive ability of biomarkers in relation to the short-term prognosis of PANSS dynamics against the background of therapy correction. Another alternative may be to study the predictive ability of biomarkers in the long-term dynamics of PANSS (1–3 years) without therapy correction (in remission). Research in this direction may improve understanding of the progression of SSD.

## 5 Conclusion

The need for more intensive investigation of item-level data from psychiatric rating scales using FA, as well as of the role of factor scores as indicators of therapeutic response in psychiatry, is highlighted. The proposed factor model of PANSS symptom dynamics during treatment in SSD will be examined in future studies using confirmatory FA to validate the structure of symptom regression.

The influence of the baseline level of LE activity on the reduction of positive symptom confirms the role of neuroinflammation in the SSD pathogenesis. The role and place of AD, AK, and anticholinergic load in the treatment of SSD require further study. The observation of a stronger therapeutic response in women with SSD should be corroborated in subsequent studies and, once confirmed, taken into consideration when prescribing treatment regimens.

Overall, this work underscores the value of routine clinical data and introduces an approach that leverages these data for the generation of research hypotheses - facilitating the translation of information from everyday clinical practice into scientific inquiry, followed by empirical verification and re-translation of the resulting into real-world clinical care. This iterative process is aimed at the continuous refinement and progressive personalization of treatment in SSD.

## Data Availability

The original contributions presented in the study are included in the article/supplementary material. Further inquiries can be directed to the corresponding author.
